# Supplementary material for: Comparison of Short- and Long-Term Mortality in Patients with or without Cancer Admitted to the ICU for Septic Shock: A Retrospective Observational Study
Source: Cancers (Basel). 2022 Jun 29;14(13):3196. doi: 10.3390/cancers14133196 (PMC9264783; doi:10.3390/cancers14133196)
Supplement: Supplementary file 1 [file cancers-14-03196-s001.zip › cancers-1789354-supplementary.pdf]

Table S1. Primary origin of malignancies in the study population.

Primary origin	n (%)
Solid tumours (n=70)	
Gastrointestinal tract	27 (38.6)
Urinary tract	14 (20.0)
Breast and gynecologic cancers	13 (18.6)
Lung	7 (10.0)
Head and neck cancers	5 (7.1)
Sarcoma	2 (2.9)
Other	2 (2.9)
Hematological malignancies (n=51)	
Leukemia	28 (54.9)
Lymphoma	19 (37.3)
Myeloma	4 (7.8)

Footnotes: Data are all expressed in median n/N (%) where n/N is the total number of patients with available data.

Table S2. Microbiological data according to subgroups.

Pathogens	*Non-cancer patients (n=91 stays)*	*Solid tumors (n=79 stays)*	*Hematological malignancies (n=69 stays)*
Gram negative	35 (38.5)	28 (35.4)	28 (40.6)
Gram positive	11 (12.1)	8 (10.1)	6 (8.7)
Virus	9 (9.9)	8 (10.1)	15 (21.7)
Fungi	14 (15.4)	13 (12.7)	19 (27.5)
Negative cultures	22 (24.2)	28 (35.4)	32 (46.4)

Footnotes: Data are all expressed in median n/N (%) where n/N is the total number of stays with available data.

Table S3. Multivariate analysis of factors associated with cancer status in patients with septic shock.

Demographics	Odds Ratio	95%CI	p value
Age (years)	0.93	[0.90 - 0.97]	**<0.01***
Male	1.23	[0.46 - 3.30]	0.68
**Other data**			
SAPS II	1.02	[0.99-1.05]	0.29
Catecholamines	1.12	[0.10-12.28]	0.92
Mechanical ventilation	0.28	[0.07-1.11]	0.07
Duration of mechanical ventilation	0.98	[0.91-1.04]	0.50
Renal replacement therapy	0.34	[0.08-1.42]	0.14
DIC	2.91	[0.75-11.26]	0.12
Transfusion	4.86	[1.47-16.04]	**<0.01***
**Outcome**			
In-hospital LOS	1.01	[1.00 - 1.03]	0.14
ICU LOS	1.01	[0.97 - 1.05]	0.51
Mortality at d28	0.42	[0.12 - 1.49]	0.18
ICU mortality	1.55	[0.45 - 5.31]	0.49
In-hospital mortality	1.48	[0.45 - 4.83]	0.52

Footnotes: * p <0.05. Abbreviations: d28, day 28 of hospitalization; DIC, disseminated intravascular coagulation; ICU, intensive care unit; LOS, length of stay; SAPS II, simplified acute physiology score II.

Table S4. Medico-economic data compared between septic shock patients with and without cancer.

Medico-economic data	Non-cancer patients n=91 stays	Cancer patients n=148 stays	p value
SAPS II	51.0 [45.0-67.0]	56.0 [42.0-68.0]	0.63
ICU Length of stay (days)	6.0 [3.0-15.0]	5.0 [2.0-11.3]	0.27
Social Security (SS) retribution per ICU stay (€)	7718 [2752- 15053]	6742 [2463-14831]	0.67
Social Security retribution per day of ICU stay (€)	1213 [1056-1764]	1243 [1017-1780]	0.98
In-hospital LOS (days)	19.5 [10.8-41.0]	25.5 [13.8-42.0]	0.33
SS retribution per hospital stay (€)	12020 [7902-24294]	18802 [9389-32551]	**0.01***
SS retribution per day of hospital stay (€)	1138 [671-1625]	1035 [718-1628]	0.76

Footnotes: Data are expressed in median [IQR 25-75] or n/N (%) where n/N is the total number of stays with available data. Abbreviations: €, euros; ICU, intensive care unit; LOS, length of stay; SAPS II, simplified acute physiology score II; SS, social security.
